# A single-channel mechanism for pharmacological potentiation of GluN1/GluN2A NMDA receptors

**DOI:** 10.1038/s41598-017-07292-8

**Published:** 2017-07-31

**Authors:** Divyan A. Chopra, Kiran Sapkota, Mark W. Irvine, Guangyu Fang, David E. Jane, Daniel T. Monaghan, Shashank M. Dravid

**Affiliations:** 1 Department of Pharmacology, Creighton University, Omaha, NE 68178 United States; 2 Department of Pharmacology and Experimental Neuroscience, University of Nebraska Medical Center, Omaha, NE 68198 United States; 3 School of Physiology & Pharmacology, University of Bristol, Bristol, BS8 1TD UK

## Abstract

NMDA receptors (NMDARs) contribute to several neuropathological processes. Novel positive allosteric modulators (PAMs) of NMDARs have recently been identified but their effects on NMDAR gating remain largely unknown. To this end, we tested the effect of a newly developed molecule UBP684 on GluN1/GluN2A receptors. We found that UBP684 potentiated the whole-cell currents observed under perforated-patch conditions and slowed receptor deactivation. At the single channel level, UBP684 produced a dramatic reduction in long shut times and a robust increase in mean open time. These changes were similar to those produced by NMDAR mutants in which the ligand-binding domains (LBDs) are locked in the closed clamshell conformation by incorporating a disulfide bridge. Since the locked glutamate-binding clefts primarily contributes to receptor efficacy these results suggests that UBP684 binding may induce switch in conformation similar to glutamate LBD locked state. Consistent with this prediction UBP684 displayed greater potentiation of NMDARs with only the GluN1 LBD locked compared to NMDARs with only the GluN2 LBD locked. Docking studies suggest that UBP684 binds to the GluN1 and GluN2 LBD interface supporting its potential ability in stabilizing the LBD closed conformation. Together these studies identify a novel pharmacological mechanism of facilitating the function of NMDARs.

## Introduction

NMDA receptors (NMDARs) are ionotropic glutamate receptors widely expressed at central excitatory synapses and elsewhere. These receptors have critical roles in normal CNS function and in neuropathological disorders. NMDARs’ distinctive physiological properties (voltage-dependency, Ca^2+^ permeability, slow onset/offset) enable their essential roles in multiple processes such as experience-dependent plasticity and learning^1^. These properties, however, also contribute to their ability to cause cell death in various neuropathological conditions when over-activated and to cause symptoms of schizophrenia when under-activated. Consequently, modulators that can alter NMDAR function have considerable potential for treating various neurological and neuropsychiatric conditions. However, development of drugs acting at NMDARs has poorly translated into the clinical setting, primarily due to unwanted side effects. NMDARs are heterotetrameric complexes composed of two glycine-binding GluN1 subunits and two glutamate-binding GluN2 subunits of which there are four subtypes (GluN2A-D) and sometimes incorporating a GluN3A or GluN3B subunit. Initial drug development focused on competitive agents, channel-blockers, and GluN2B-selective negative allosteric modulators (NAMs)^1, 2^. Recent drug development efforts have focused on NAMs with other patterns of subtype-selectivity and on positive allosteric modulators (PAMs) to potentiate NMDAR function^3, 4^.

The development of PAMs for NMDARs has gained significant interest since NMDAR potentiation is expected to be useful in treating schizophrenia and cognitive deficits^3–6^. Promising drug-like PAMs with varied patterns of subunit-selectivity have been identified^7–13^. However, the gating mechanism of these modulators remains poorly understood. We have recently identified UBP684 as a highly effective potentiator of all GluN1/GluN2 subtypes with a similar degree of potentiation at each subtype^14^. UBP684 is the naphthoic acid homologue of the phenanthroic acid compound UBP646^7^. Compounds in this family, including UBP684, are allosteric modulators displaying either potentiating or inhibiting activity at NMDARs. They do not substitute for either L-glutamate or glycine and they do not activate the receptor nor act as competitive antagonists or voltage-dependent channel blockers^7^. The activity of these compounds is retained in receptors with the N-terminal domain deleted. In chimeric experiments, the PAM activity was found to correspond to the S2 domain of the GluN2 subunit whereas negative allosteric activity correlated to the GluN2’s S1 domain^7^. Because UBP684 robustly potentiates NMDAR responses, we selected this compound for single channel mechanistic studies.

Here we have determined the effect of UBP684 on NMDAR function on heterologously expressed GluN1/GluN2A receptors. We utilized previously known changes in gating mechanisms identified by mutational analysis to evaluate the potential conformational change induced by UBP684. Our studies suggest a novel mechanism of pharmacological potentiation of NMDARs wherein the ligand-binding domain (LBD) of the GluN2 subunit is stabilized in a closed, agonist-bound conformation.

## Results

### Effect of UBP684 on macroscopic currents is dependent on intracellular milieu

**Figure 1:**
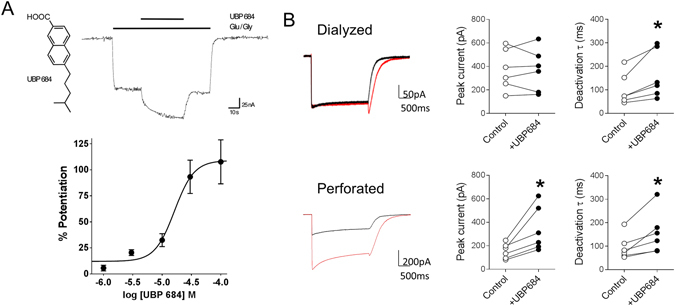
UBP684 potentiates GluN1/GluN2A receptor responses and slows their deactivation kinetics. (**A**) UBP684 structure and PAM activity on GluN1/GluN2A receptors expressed in Xenopus oocytes. After obtaining a stable steady-state response to 10 µM L-glutamate and 10 µM glycine, application of 50 µM UBP684 potentiates the agonist response (upper panel). Dose-response of UBP684 potentiation of GluN1/GluN2A receptor responses under saturating agonist conditions (300µM L-glutamate/300 µM glycine) is shown in the lower panel. (**B**) Whole-cell recordings (left panel) were obtained from HEK293 cells either in dialyzed (top row) or perforated mode (bottom row). Individual cell peak current and deactivation time in response to UBP684 application (right two panels). Using the fast concentration-jump technique, cells were exposed to a solution containing no drug and switched to a solution containing glutamate and glycine (black traces) or glutamate, glycine and UBP694 (red traces). UBP684 (100 µM) potentiated glutamate (100 µM) and glycine (100 µM) induced peak currents in the perforated condition, but not in the dialyzed condition. Slowing of the deactivation rate by UBP684 was observed under both modes. N = 6, *P < 0.05, paired *t*-test.

We first measured the EC_50_ of UBP684 in oocytes expressing GluN1/GluN2A subunits. UBP684 produced a maximal potentiation of 107 ± 21% with a EC_50_ of 28 ± 12 µM. Using fast concentration-jump experiments, we tested the effect of UBP684 on macroscopic GluN1/GluN2A whole-cell currents expressed in HEK293 cells under dialyzed (non-perforated) conditions. UBP684 (100 µM) was co-applied with glutamate (100 µM) and glycine (100 µM) (Fig. 1). UBP684 produced no effect on the peak response (p = 0.9327, N = 6, paired t-test, I_UBP684_/I_control_ = 0.996 ± 0.077) but slowed the deactivation kinetics from 103.08 ± 27.63 ms in control condition to 163.53 ± 41.58 ms (p = 0.0226). A transient rise in current was observed in the whole-cell recordings when the solution containing UBP684 and agonists was washed-out which is similar to effect produced by pregnenolone sulfate^15, 16^. We and others have shown that modulation of NMDAR responses by the endogenous allosteric modulator pregnenolone sulfate is affected by the mode of whole-cell recording^16–18^. Thus we performed perforated whole-cell recordings using gramicidin to test whether keeping the intracellular milieu intact would affect UBP684 modulatory actions. In perforated patch mode, UBP684 significantly increased the peak response by two-fold (I_UBP684_/I_control_ = 2.134 ± 0.204, p = 0.0207, N = 6,) and also slowed deactivation from 95.29 ± 21.31 ms to 156.62 ± 36.54 ms (p = 0.0206). Additionally, a transient rise in whole-cell current was observed during drug wash-out. Thus the increase in deactivation kinetics and transient increase in current during wash-out was independent of the whole-cell mode. UBP684 alone did not have any effect on baseline of HEK293 cells expressing GluN1/GluN2A receptors (N = 3). We also conducted fast jump experiments in perforated whole-cell mode where glycine ± UBP684 were present in control solution and the cell was moved from control solution to one containing glycine and glutamate ± UBP684. We found that under these conditions UBP684 produced an increase in peak currents (I_UBP684_/I_control_ = 1.413 ± 0.077, N = 5) as well as slowed the deactivation kinetics from 88.0 ± 20.1 ms to 112 ± 21.5 ms (paired *t*-test, p = 0.0069).

### UBP684 produces dramatic changes in the shut times which resemble effects of locked closed conformation of GluN2 ligand-binding domain

**Figure 2:**
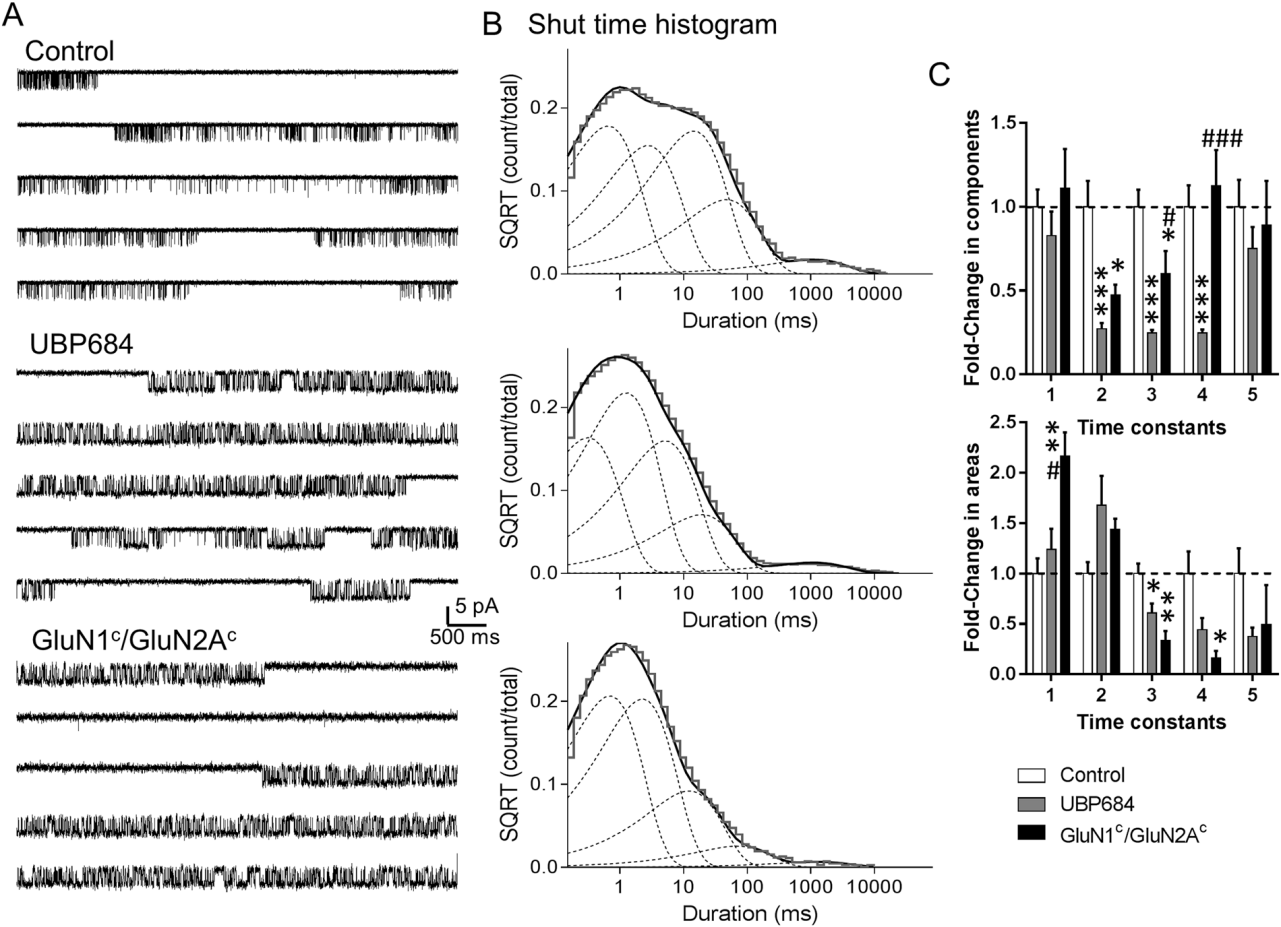
UBP684 induced changes in channel gating resemble effect of locking ligand-binding domain in closed conformation. Cell-attached patches containing one active channel were obtained with agonists alone (glutamate and glycine) (N = 7) or agonists plus UBP684 (N = 7) or from mutant GluN1c/GluN2Ac (N = 4) where the LBD was locked closed by engineered cysteine bridges. (**A**) Representative single-channel traces are shown. Single-channel recordings were filtered at 5 kHz (2 kHz for presentation) and digitized at 20 kHz. (**B**) Shut time histograms were fitted with 5 exponential components. Fitting of composite shut time histograms is shown. Individual shut time histograms were fitted and the changes in time constants and their areas were compared. The fold change in the normalized time constants and area of time constants is shown. Data is compared using one-way ANOVA followed by Tukey’s post-hoc analysis. *P < 0.05, **P < 0.01 and ***P < 0.001 compared to control. ^#^P < 0.05, ^###^P < 0.001 compared to UBP684. SQRT stands for square root.

**Figure 3:**
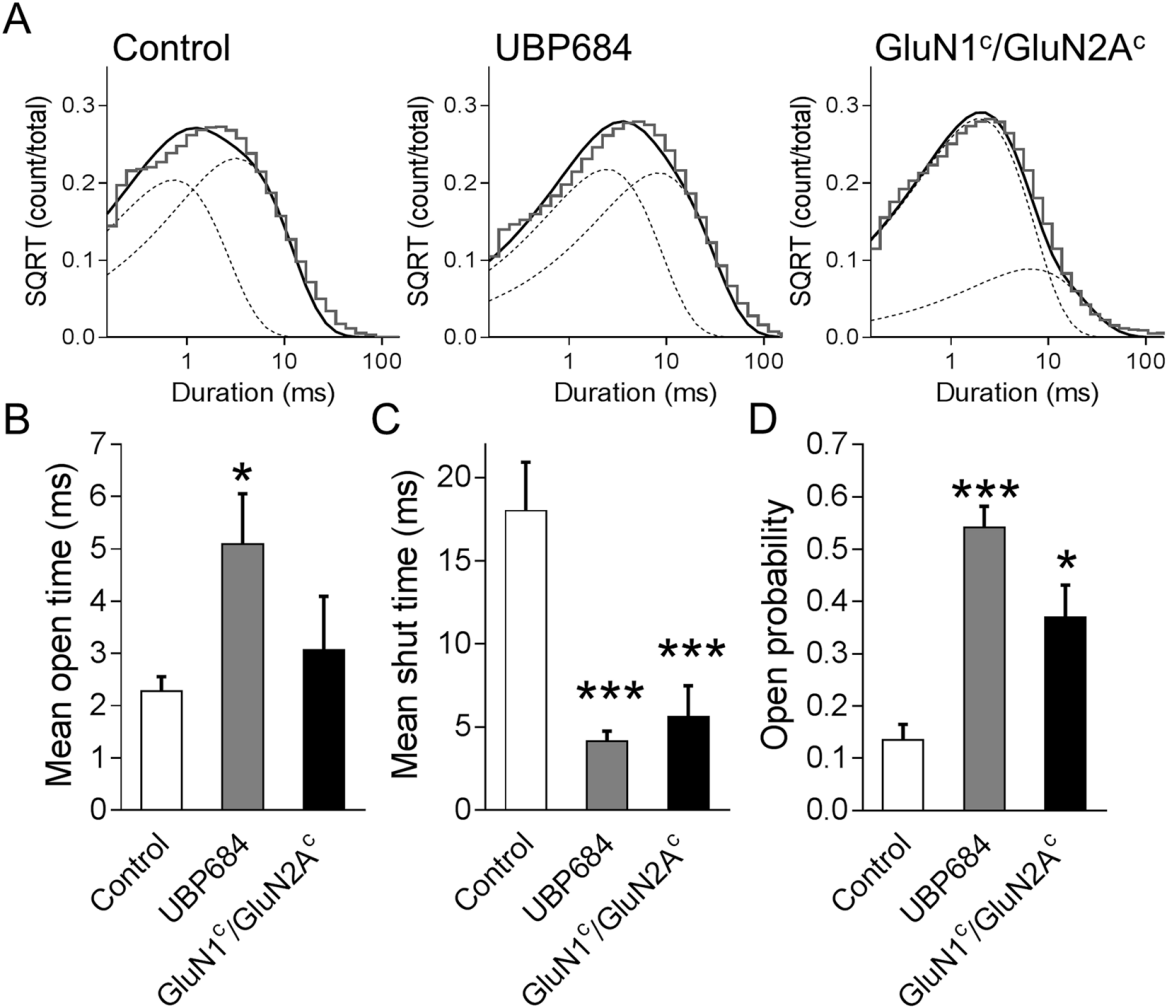
UBP684 robustly affects the mean open time in addition to its effects on shut states. (**A**) Open time histograms were fitted with 2 exponential components. Fitting of composite open time histograms is shown. Individual open time histograms were fitted and the time constants and areas are presented in Table 1. UBP684 (N = 7) increased (**B**) the mean open time and reduced (**C**) the mean shut time and lead to an overall increase in (D) open probability compared to control (N = 7). The GluN1c/GluN2Ac mutant (N = 4) shared the reduction in mean shut time and overall increase in open probability but did not lead to a significant increase in mean open time. *P < 0.05 and ***P < 0.001. SQRT stands for square root.

To determine the mechanism by which UBP684 exerts its potentiating effects, we studied the effect of UBP684 on single-channel responses in the cell-attached patch clamp mode to replicate the gramicidin-perforated whole-cell mode as it has minimal effect on intracellular milieu. Single-channel data obtained from one active channel was analyzed and fitted to kinetic models based on previous analysis of GluN1/GluN2A single channel activity^19–22^ (Fig. 2). Both control and UBP684 patches demonstrated reasonably good fits with a scheme with five shut states and two open states as previously described^23^ suggesting this model provides a reasonable description of the receptor gating. These results demonstrate that UBP684 does not affect the number of kinetic states that the receptor traverses before opening. Analysis of shut time constants revealed a dramatic shift in closed conformational states by UBP684. The time constants τ2, τ3 and τ4 were significantly reduced by UBP684 (Table 1). UBP684 also caused an increase in the area of τ2 while reducing the area of the τ3, τ4 and τ5 shut time constants (Table 1). The shift in the shut time constants is in accordance with a reduction in the mean shut time in the presence of UBP684 (Fig. 3). Interestingly, such a dramatic shift in shut time has been observed previously by mutant receptors in which the LBDs are covalently kept in a closed conformation by disulfide bridges of engineered cysteines^22, 24^. In addition such effects are also produced by application of DTT which may also stabilize the closed conformation of the LBD^25^.

**Table 1:** Time constants and area of the open and shut time histogram fits with Hidden Markov models.

Time constants (ms) and areas (%)	Condition		
Open time	Control (N = 7)	UBP684 (N = 7)	NR1^c^/NR2A^c^ (N = 4)
τ1	0.26 ± 0.03	0.86 ± 0.24*	0.73 ± 0.19*
τ2	2.76 ± 0.29	5.88 ± 1.08*	2.40 ± 0.25
a1	20 ± 3	16 ± 5	43 ± 17
a2	80 ± 3	84 ± 5	56 ± 17
**Shut time**			
τ1	0.51 ± 0.05	0.42 ± 0.07	0.57 ± 0.12
τ2	5.06 ± 0.78	1.37 ± 0.18***	2.40 ± 0.32*
τ3	18.7 ± 1.9	4.63 ± 0.4***	11.3 ± 2.52*
τ4	62.3 ± 7.9	15.4 ± 1.3***	70.2 ± 13.2
τ5	1189 ± 190	895 ± 151	1060 ± 314
a1	22 ± 3	27 ± 4	48 ± 5**
a2	25 ± 3	43 ± 7*	36 ± 3*
a3	42 ± 4	26 ± 4*	14 ± 4**
a4	10 ± 2	4 ± 1*	1.7 ± 1*
a5	0.5 ± 0.1	0.2 ± 0.04*	0.2 ± 0.1

The histograms were fitted with a model containing 5 closed states and 2 open states. Mean ± SEM are presented. Comparison was performed using unpaired *t*-test in relation to control. *P < 0.05, **P < 0.01 and ***P < 0.001.

To directly compare the effects of UBP684 to the effects of having the LBD constrained in the closed conformation in our experimental conditions, we studied the channel kinetics of single active channels from mutant NMDARs with cysteines introduced at N499C and Q686C in GluN1 (GluN1^c^) and K487C and N687C in GluN2A (GluN2A^c^) (Fig. 2) as previously described^22^. Several changes in the shut time constants and the areas of shut time were similar in the cysteine-substituted NMDARs and UBP684-potentiated NMDARs. ANOVA analysis of normalized time constants and area revealed similarities in the effect of UBP684 and mutant in reducing τ2 and τ3 and area of τ3 compared to control. Based on previous kinetic modeling of NMDAR activation these time constants most likely represent changes in the GluN1 and GluN2 conformations (upon agonist binding) respectively before channel opening^26, 27^. Thus both UBP684 and locked-ligand binding domain receptor have strong effects on these and in particular τ3. Additionally, some differences were noted between UBP684 and GluN1^c^/GluN2A^c^ mutant. The most noteworthy difference among the two conditions is the change in τ4 which represents a fast desensitized state. Thus it appears that UBP684 also affects desensitization of the receptor. Accordingly, UBP684 affected the rate constants for receptor traversing the desensitized states (Table 2).

**Table 2:** Hidden Markov maximum interval likelihood fitting of the steady state currents.

Rates (s^−1^)	Condition		
	Control N = 7	UBP684 N = 7	NR1^c^/NR2A^c^ N = 4
*C*_*1*_ → *C*_*2*_	110 ± 20	350 ± 20***	145 ± 35
*C*_*2*_ → *C*_*1*_	80 ± 30	220 ± 45*	95 ± 25
*C*_*2*_ → *C*_*3*_	360 ± 60	1010 ± 160**	505 ± 85
*C*_*3*_ → *C*_*2*_	1030 ± 125	930 ± 245	615 ± 225
*C*_*3*_ → *O*_*1*_	700 ± 60	1330 ± 140**	1345 ± 200**
*O*_*1*_ → *C*_*3*_	1495 ± 185	530 ± 85***	1040 ± 235
*O*_*1*_ → *O*_*2*_	1605 ± 370	825 ± 290	590 ± 425
*O*_*2*_ → *O*_*1*_	1075 ± 150	730 ± 110	735 ± 230
*C*_*1*_ → *D*_*1*_	1.7 ± 0.4	2.8 ± 0.7	1.9 ± 0.8
*D*_*1*_ → *C*_*1*_	1.0 ± 0.1	2.3 ± 0.8	1.3 ± 0.4
*C*_*2*_ → *D*_*2*_	11.8 ± 5.0	54.7 ± 11**	9.7 ± 4
*D*_*2*_ → *C*_*2*_	21.8 ± 4.5	49.2 ± 11*	15.9 ± 2

**Figure 4:**
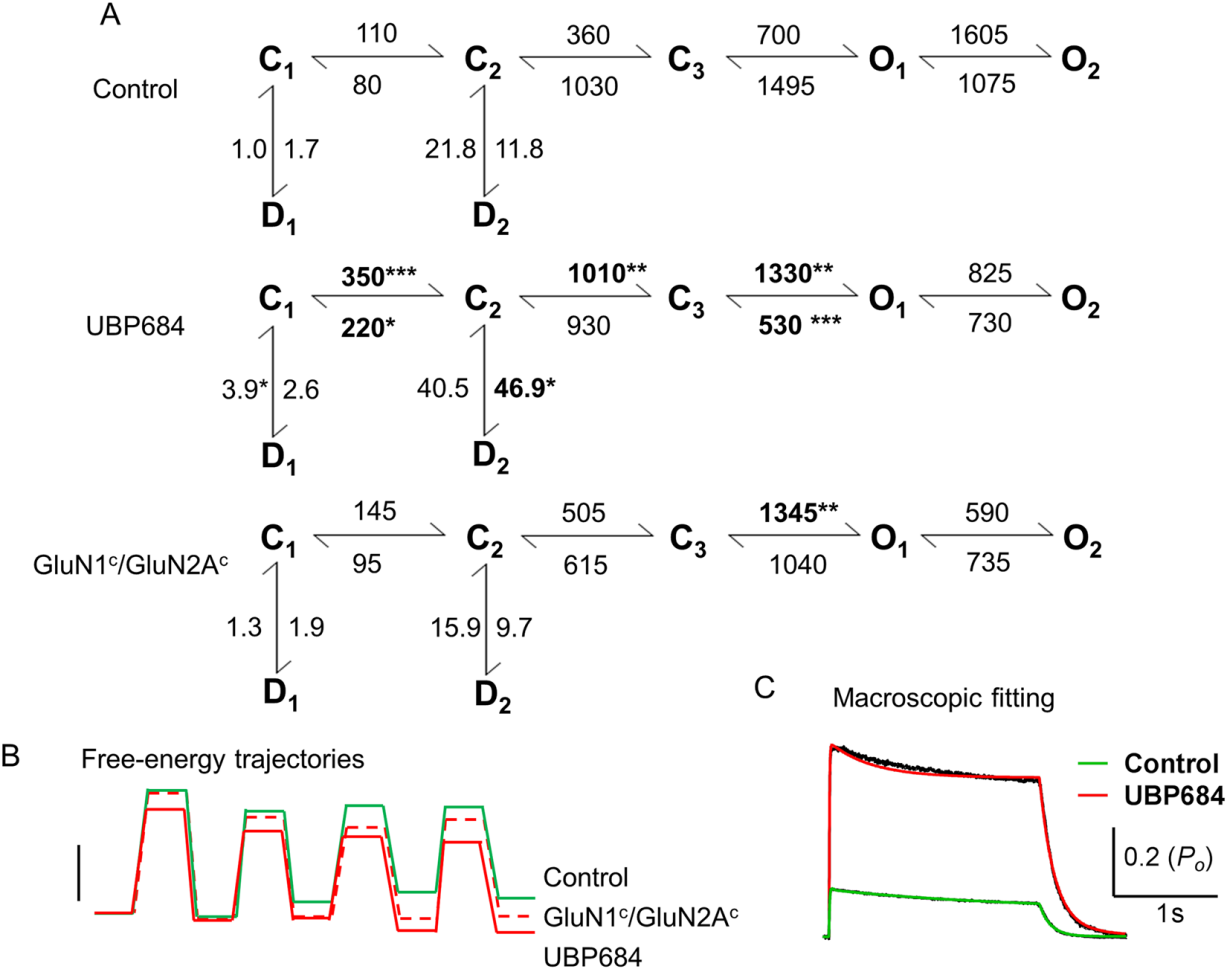
Kinetic mechanism describing the effects of UBP684 on GluN1/GluN2A receptor activation. (**A**) MIL fit of single-channel data to assess the gating mechanism underlying potentiation of UBP684 is shown. All rates are in sec^−1^. Bold numbers with asterisks denote the rates which were significantly different from glutamate/glycine control patches. Rates ± SEM are presented in Table 2. Data was analyzed using unpaired *t*-test. (**B**) Free-energy trajectories for the kinetic states in the different models are presented. The free energies of the active states for the mutant GluN1c/GluN2Ac is lower than control but is still higher than UBP684. Scale bar represents 1 k_B_T. (**C**) Macroscopic current profiles were obtained by whole-cell recordings (black traces). Cells were equilibrated to glycine ± UBP684 before exposing to glycine + glutamate ± UBP684 as detailed in Methods. Current profiles were fitted to the kinetic Markov models for control (green) and UBP684 (red) that included glutamate binding and unbinding steps. Except the desensitization rates and the agonist binding and unbinding rates all other rates were fixed to those depicted in the kinetic models. The models were able to accurately predict the activation and deactivation kinetics. Rates obtained from least squares fitting are presented in Table 3.

Idealized current records were fitted to the gating scheme as described in Fig. 4. All rates have units of s^−1^. Data are mean ± SEM from patches containing one active channel fitted individually. The rates were compared by unpaired *t*-test. *P < 0.05, **P < 0.01 and ***P < 0.001.

To further understand the commonality between the actions of UBP684 and GluN1^c^/GluN2A^c^ mutant we assessed the effect of these manipulations on mean open time, mean shut time and open probability. As seen in Fig. 3, UBP684 robustly increases mean open time, reduces mean shut time and increases overall open probability. UBP684 increased the mean open time (±SEM) from 2.27 ± 0.28 ms (262,707 events; N = 7) in control patches to 5.09 ± 0.96 ms (457,992 events; N = 7, p = 0.0158, unpaired *t*-test). The mean shut time was reduced by UBP684; 20.05 ± 2.92 ms in control patches (263,098 events) to 4.13 ± 0.61 ms in UBP684 patches (458,009 events, p < 0.001). Consequently, the open probability, measured over the entire length of recordings, was found to increase from 0.135 ± 0.030 in control patches to 0.542 ± 0.033 in UBP684 patches (p < 0.001). The amplitude of openings was unaffected by UBP684. The GluN1^c^/GluN2A^c^ mutant was found to significantly lower mean shut time compared to control (5.62 ± 1.86 ms, p = 0.0158, unpaired *t*-test; 135,773 events; N = 4) and increase open probability (0.370 ± 0.062, p < 0.001; Fig. 3), while the mean open time of the mutant receptor responses is between responses in the control and UBP684 conditions. The open time histogram fits indicated that the open time constant τ1 was significantly higher than control in both the UBP684-treated and the GluN1^c^/GluN2A^c^ mutant conditions (Table 1). There was also a shift in the area of time constants observed in the GluN1^c^/GluN2A^c^ mutant but this did not reach significance (Table 1).

Further maximum interval likelihood (MIL) fitting of single-channel data indicated that UBP684 affected several gating steps to produce potentiation (Fig. 4). The slower gating steps were affected showing an increase in the forward rate constants, driving the receptor faster into the open states. This is in accordance with the changes seen with shut-time constants τ2 and τ3. The faster gating steps were also affected, keeping the receptor for a longer time in the open states, as seen with an increase in the open time constants τ1 and τ2. The changes in slower rate constants represent a larger conformational change in the LBD of the GluN2 subunit^21, 27^. The faster rate constants may represent a gating mechanism that occurs near the pore of the ion-channel^28^. The faster recovery from the desensitized states (D2-C2) implies that the receptor spends less time in the long-lived desensitized state. The rate constants obtained from MIL analysis were also able to satisfactorily predict the macroscopic wave forms for control and UBP684 (Fig. 4; Table 3) suggesting that the kinetic models predict channel behavior. Interestingly, our experimental data shows a small but significant increase in glutamate potency (Table 3) which may explain the slower deactivation. However, the macroscopic fitting suggests no change in apparent glutamate affinity and in fact a small decrease in glutamate affinity (Table 3). Additionally, simulations using the gating rates from MIL fits depicted in Fig. 4 with the same agonist binding and unbinding rates for control and UBP684 suggest that these models are sufficient to produce slower deactivation rate without shift in apparent agonist affinity. Thus one possibility is that differences in the gating of agonist bound states may underlie the slower deactivation in the presence of UBP684 in addition to potential changes in agonist affinity.

**Table 3:** Fitting of macroscopic GluN1/GluN2A current response and effect of UBP684.

Rates	Experiment		Macroscopic Fit	
	Control	UBP684	Control	UBP684
*b*+	—	—	30	37
*b−*	—	—	25	84
*d1*+	—	—	1.00	8.2
*d1−*	—	—	0.30	2.0
*d2*+	—	—	22	165
*d2−*	—	—	46	107
Residual	—	—	2.67 × 10^−6^	8.85 × 10^−5^
*glutamate EC*_*50*_ *or K*_*d*_	4.62 ± 0.32	3.12 ± 0.52	0.84	2.2
*P*_*O*_ (*peak*)	0.54 ± 0.04	0.13 ± 0.03	0.55	0.13
*MOT*	2.276	5.093	2.1	0.453

The normalized residual sum of squares (indicated as Residual) were derived from the macroscopic fitting with the gating rates fixed to those derived from MIL fits (Fig. 4); the desensitization (*d*) and agonist binding (*b*+) and unbinding (*b−*) rates were set as free parameters. The agonist binding rates are in µM^−1^ s^−1^ and all other rate constants are s^−1^. The *K*_*d*_ is in µM and calculated as *b*−/*b*+. The desensitization rates were constrained to 5-fold ± rates derived from MIL fits (Fig. 4). Open probability (*P*_*o*_), mean open time (MOT).

In comparison to the previous analysis of GluN1^c^/GluN2A^c^ mutant^22^ we found similarity in the overall shift in the exponential components to left in the GluN1^c^/GluN2A^c^ compared to control. Similar effects are also noted for UBP684. However, there are differences in how this mutant affects time constants and gating rates in the two studies. This may arise due to differences in the control channel properties. For example, we found a lower mean open time of channels resulting in lower open probability which may explain differences in rate constants. Differences in pH, EDTA concentration and/or other constituents of the pipette solution may explain these differences in channel behavior.

### UBP684 potentiation is dependent upon GluN2 conformational flexibility

**Figure 5:**
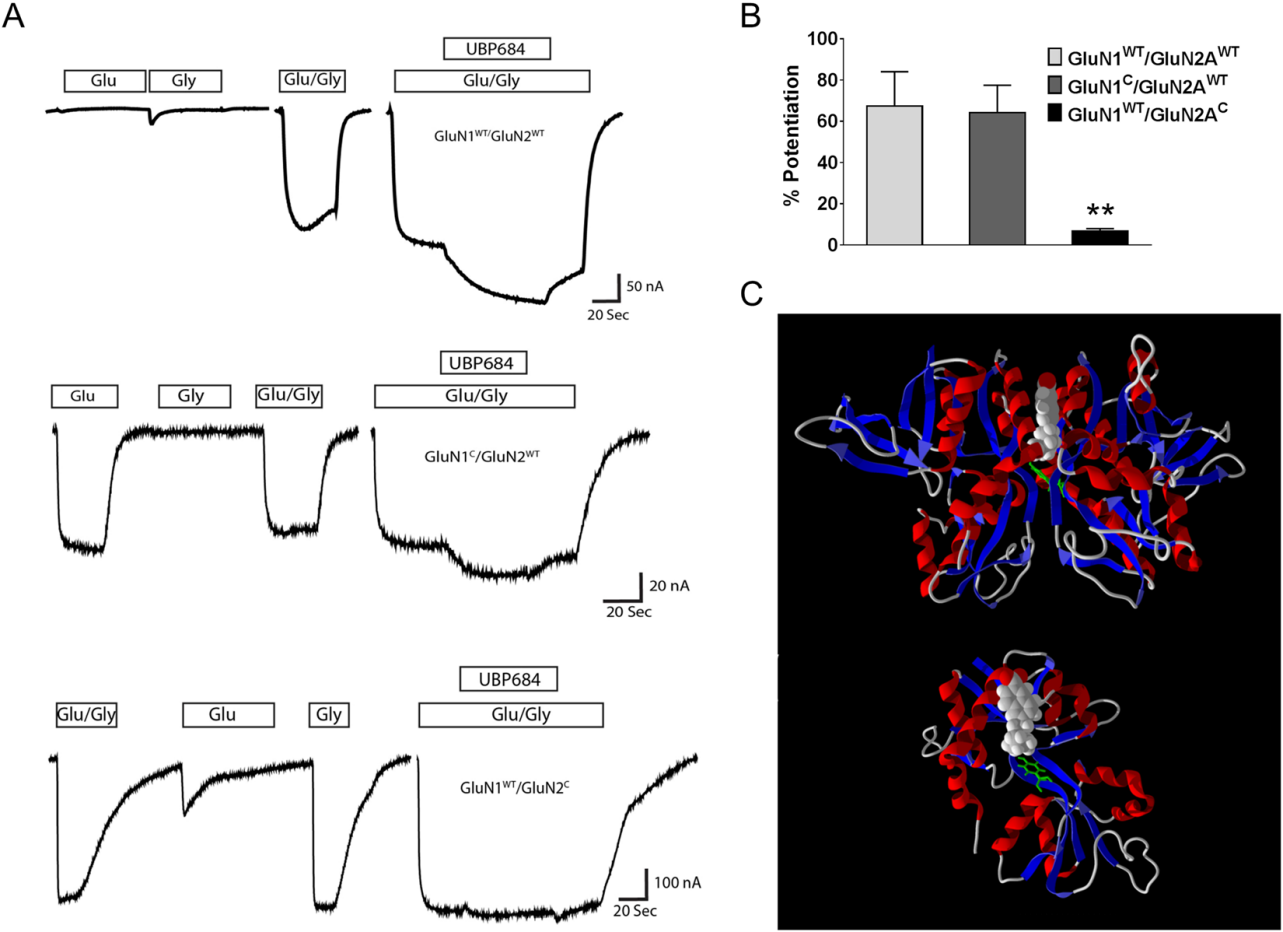
UBP684 potentiation is dependent on GluN2 LBD flexibility. (**A**) Current traces showing the effect of UBP684 on wildtype, GluN1 LBD-locked (GluN1^C^/GluN2^WT^) or GluN2 LBD-locked (GluN1^WT^/GluN2^C^) receptors. (**B**) UBP684 potentiated wildtype and GluN1 LBD-locked receptors but not GluN2 LBD-locked receptors (one-way ANOVA followed by Tukey’s test; P = 0.0012 and 0.0093 for GluN1^WT^/GluN2A^c^ compared to GluN1^WT^/GluN2A^WT^ and GluN1^C^/GluN2A^WT^ respectively; N = 19–32). (**C**) Molecular modeling of UBP684 binding to the GluN1/GluN2A receptor LBD dimer. Top: UBP684 (space filled) is shown docked into the GluN1/GluN2A LBD intersubunit interface. Modeling suggests that the carboxylic acid group of UBP684 interacts with positively charged residues on the top of the LBD away from the transmembrane domains and near the N-terminal domains. The alkyl side chain terminates near the LBD hinge region near GluN1 Y535 shown in green. Bottom: The same docking of UBP684 is shown rotated 90° in the horizontal plane with the GluN1 LBD removed and GluN1’s Y535 shown for reference.

The LBD locked single-channels studies by Kussius and Popescu^22^ have demonstrated that GluN2 LBD-locked receptor open at higher efficacy and resembles a receptor with both GluN1 and GluN2 in LBD-locked conformation. Thus the observed UBP684-induced changes in single channel closed states are consistent with UBP684 potentiating by promotion of a conformation resembling or mimicking GluN2 LBD-locked state. To evaluate a possible difference between GluN1 and GluN2 conformational changes contributing to UBP684 potentiation, GluN1^c^/GluN2A^WT^ and GluN1^WT^/GluN2A^c^ were expressed in Xenopus oocytes and the NMDAR response to UBP684 was determined. As previously reported^22^, GluN1^c^/GluN2A^WT^ receptors gave responses to L-glutamate applied alone that were comparable to the application of glycine plus L-glutamate. Glycine by itself produced negligible responses. Conversely, GluN1/GluN2A^c^ receptors displayed a full response to glycine applied alone, but not with L-glutamate alone. As shown in Fig. 5, UBP684 caused a significantly higher potentiation of GluN1^c^/GluN2A^WT^ receptor responses (64 ± 13) than those of GluN1^WT^/GluN2A^c^ receptors (7 ± 1) (one-way ANOVA followed by Tukey’s test; p = 0.0012 and 0.0093 for GluN1^WT^/GluN2A^c^ compared to GluN1^WT^/GluN2A^WT^ and GluN1^C^/GluN2A^WT^ respectively; N = 19–32).

## Discussion

### UBP684 a novel potentiator of the GluN1/GluN2A NMDARs

Recent studies have identified multiple, novel classes of allosteric modulators of NMDARs. These include both NAMs and PAMs with some that exhibit subtype selectivity. We used a novel PAM, UBP684 which is the naphthene homolog of the phenanthrene PAM UBP646^7^. UBP684 increased whole-cell current responses mediated by GluN1/GluN2A by approximately two-fold. This effect is dependent on the patch clamp recording mode used (perforated, not dialyzed). This finding is similar to our previous observation with pregnenolone sulfate^16^. It will be of interest to study whether receptor phosphorylation or other signaling pathways that can affect receptor function/conformation underlie this effect. It will be also of interest whether other potentiators produce similar intracellular milieu-dependent effects on channel properties. UBP684 slowed the deactivation rate of the receptor suggesting a potential difference in agonist interaction with the receptor. Under steady state conditions, the increase in open probability induced by UBP684 can be accounted for by both an increase in the mean open time of the receptor and a reduction in the mean shut time. The rightward shift in the open time constants by UBP684 (Fig. 3) as seen with the exponential fitting of the open time histograms suggest that UBP684 stabilizes the receptor’s open state for a longer duration in both the short-lived and long-lived open states accounting for the increase in mean open time. In addition, in the shut-time histograms with five exponential components, there is a dramatic leftward shift with the shoulder (or second peak in overall fitting seen with the control histogram, Fig. 2) significantly abolished in the presence of UBP684. This corresponds to greatly reduced τ2, τ3 and τ4 long-lived shut-time constants. Based on a previous model of NMDAR gating^21, 26, 27^, the reduction in the longer shut-time constants or a slower conformational change is suggestive of a role of GluN2 subunit in the potentiation seen by UBP684.

### Gating effects of UBP684 in comparison to earlier potentiators and mutants

Previously analyzed potentiators of NMDARs have been found to enhance the efficacy of the receptor by either increasing the mean open time or by having subtle effects on the shut time components^9, 16^. In comparison, the dramatic leftward shift in shut time components induced by UBP684 is unique. To better understand the effect of UBP684, single-channel recordings using the GluN1^c^/GluN2A^c^ mutants were carried out. The LBD of the mutants were locked in a closed conformation, as if agonist was continuously bound^22, 24^. The shut-time histograms of this mutant receptor showed similarity with the histogram seen with UBP684, wherein the long-lived shut time constants τ2 and τ3 are greatly reduced. It has been suggested that even in the presence of saturating concentrations of agonists (here glutamate), the LBD domain undergoes fluctuations between opening and closing of the cleft. Since locking of the GluN2 LBD, but not GluN1 LBD, increases receptor efficacy, these conformational changes in the cleft appear to be restricted to glutamate binding or a GluN2 gating step. In accordance with the attribution of the long-lived shut states (particularly τ3 in our case) to GluN2 conformational changes^26, 27^ there is a dramatic change in the duration and area of these states^22^. Thus, these results suggest that UBP684 influences the behavior of the receptor as if the LBD is constantly closed leading to a similarity in the shut time histogram to the LBD mutant. Using the LBD-locked mutants separately (GluN1^c^/GluN2A and GluN1/GluN2A^c^), we find that UBP684 preferentially potentiates responses in the GluN1^c^/GluN2A receptor. Thus, consistent with the single channel analysis, UBP684 appears to be enhancing conformational changes mediated by the GluN2 subunit more than that governed by the GluN1 subunit. A potential caveat is that the locked GluN2A-LBD construct may be maximally open such that potentiation by any mechanism is obscured. However, further potentiation of the GluN1^c^/GluN2A^c^ receptor’s activity can be seen by cysteine modification of receptors including GluN1 A652C which leads to maximal activation^24^. Also, the conclusion that activity of the GluN1^c^/GluN2A^c^ construct is not saturated is consistent with the greater mean open time in the presence of UBP684 than in the GluN1^c^/GluN2A^c^ construct. Since wildtype receptors with UBP684 display a longer mean open time than GluN1^c^/GluN2A^c^ receptors and yet UBP684 potentiation is largely blunted in this construct, it is possible that UBP684 promotes a state of greater efficacy than seen in GluN1^c^/GluN2A^c^ construct, but locking the LBD prevents the ability of UBP684 to promote greater efficacy.

UBP684 is likely to have additional effects since it enhances open time stability leading to longer open states (Fig. 3), changes the time constants and rates for desensitized states (Figs 2 and 4) and has more robust effects on the forward rate constants (C1-C2, C2-C3) and reverse rate constant (O1-C3) compared to GluN1^c^/GluN2A^c^ (Fig. 4). In relation to changes in desensitization rates, the location of UBP684 at the LBD interface based on docking analysis (Fig. 5) has similarity to the proposed mechanism of cyclothiazide in inhibiting AMPA receptor desensitization^29^. Additionally, this location can influence receptor conformations which are not affected by the GluN2-ligand-locked mutant. One interpretation of the changes in rate constants and free-energy trajectories may be that the GluN1^c^/GluN2A^c^ state is an intermediary state in the effects of UBP684 which accelerate the forward rates to channel opening with greater efficacy. While the PAM UBP684 appears to stabilize the open channel states, the structurally related NAM, 2-naphthoic acid, stabilizes the closed states^30^. These agents have opposite effects on mean open time as well as on mean shut time consistent with their PAM and NAM activities. They also have generally opposite effects on the time spent in the different closed states. UBP684 decreases the area of the 3 slowest shut states while increasing the area of the first two shut states and conversely 2-naphthoic acid increases the area of three longest shut states and decreases the area of the shortest shut state^30^. One of the caveats in our study is that the single channel data is correlational between the effect of UBP684 and the effect of the LBD locked mutant. Although, the testing in oocytes addresses this caveat, it will be important to directly test the loss of further change in single channel behavior when UBP684 is tested in the LBD locked mutant.

The UBP PAMs do not bind at the orthosteric glutamate or glycine binding sites, nor do they behave like channel blockers and they do not act by binding to the N-terminal domain^7^. These findings are consistent with chimeric studies which indicate that the second segment (S2) domain of the GluN2 LBD is critical for UBP-PAM activity^7^. By analogy to AMPA receptor potentiation by CZX614^31^, PAM binding at the LBD dimer interface near the hinge region of the LBD clamshell structure potentially stabilizes the agonist-bound conformation. Our molecular docking studies show that UBP684 can be docked into this space in GluN1/GluN2A subunits with the alkyl chain of UBP684 terminating close to the hinge region. This site is similar to that recently described by crystalography for GluN1/GluN2A potentiator GNE-6901^13^. It is also possible, however, that UBP684, is acting near the distal S2/S2-M3 linker region near the activation gate which is present on the M3 segment of the TMD in a manner similar to that proposed for neurosteriods^32^. UBP684 is not likely to be binding where spermine or PYD-106 bind as these involve the N-terminal domain^33, 34^ which is unnecessary for UBP-PAM activity^7^.

## Conclusion

Our results reveal a unique gating mechanism for pharmacological potentiation of NMDARs. We have used a correlational approach to predict the potential conformational changes induced by a novel GluN1/GluN2A potentiator, however further mutational and structural studies are required to identify the precise site of action of UBP684 and other PAMs which may have similar site of action. It will be interesting to test whether GNE-6901^13^ produces similar gating effects which would demonstrate a conserved mechanism across different families of potentiators. Our results define a mechanism of action for a novel class of NMDA receptor potentiators and suggest a structural basis that should facilitate further drug development of NMDAR PAMs for multiple therapeutic applications.

## Materials and Methods

### Xenopus oocyte physiology

To express NMDA receptors in Xenopus oocytes, cDNA plasmids encoding the NMDAR1a subunit (GluN1a) (generously provided by Dr. Shigetada Nakanishi, Kyoto, Japan). GluN2A (generously provided by Dr. Peter Seeburg, Heidelburg, Germany), and GluN1 and GluN2A constructs with cysteine substitutions at N499C and Q686C in GluN1 (hereafter GluN1^c^) and for K487C and N687C in GluN2A (hereafter GluN2A^c^)^22^ (generously provided by Dr. Gabriela Popescu, University of Buffalo) were linearized with Not I (GRIN1a, GRIN1^c^, GRIN2A^c^) or EcoR I (GRIN2A) and transcribed *in vitro* with T7 RNA polymerase using the mMessage mMachine transcription kits (Ambion, Austin, TX, USA).

Oocytes from mature female Xenopus *laevis* (Xenopus One, Ann Arbor, MI, USA) were removed and isolated using procedures approved by the University of Nebraska Medical Center’s Institutional Animal Care and Use Committee in compliance with the National Institutes of Health guidelines. NMDA receptor subunit RNAs were dissolved in sterile distilled H_2_O. GluN1a and GluN2 RNAs were mixed in a molar ratio of 1:1–3. 50 nl of the final RNA mixture was microinjected (15–30 ng total) into the oocyte cytoplasm. Oocytes were incubated in ND-96 solution for 1–5 days at 17 °C prior to electrophysiological assay.

Electrophysiological responses were measured using the two electrode voltage clamp model OC-725B amplifier (Warner Instruments, Hamden, Connecticut,). The recording buffer contained 116 mM NaCl, 2 mM KCl, 0.3 mM BaCl_2_ and 5 mM HEPES, pH 7.4. Response magnitude was determined by the steady plateau response elicited by bath application of 10 µM L-glutamate plus 10 µM glycine and held at a membrane potential of −60 mV. After obtaining a steady-state response to agonist application, test compounds were bath applied (Automate Scientific 16-channel perfusion system) and the responses were digitized for analysis (Digidata 1440 A and pClamp-10, Molecular Devices). Dose-response relationships were fit using GraphPad Prism, ISI Software, using a nonlinear regression to calculate EC_50_ and % maximal effect.

### Expression of recombinant NMDARs

Human embryonic kidney (HEK) 293 cells were transiently transfected with cDNA coding for GluN1 and GluN2A subunits or GluN1^c^ and GluN2A^c^ subunits^22^ and green fluorescent protein (GFP) in the ratio of 1:2:0.5 using Viafect^TM^ reagent (Promega Corporation, Madison, WI) as previously described^16^. Electrophysiology experiments were performed 16–48 hours after transfection.

### Mammalian cell electrophysiology

Whole-cell and single-channel recordings were obtained from transfected HEK 293 cells at room temperature (22–25 °C) in an external solution containing (in mM) 150 NaCl, 3 KCl, 10 HEPES, 0.02 mM EDTA and 6 mannitol. Recordings were conducted in the absence of nominal extracellular CaCl_2_. The external pH was adjusted to 7.4 with NaOH. Cells or patches were exposed to either 100 μM glutamate and 100 μM glycine (control patches) or 100 μM glutamate, 100 μM glycine and 100 μM UBP684. For whole-cell recordings, agonists and UBP684 were added to the extracellular solution. For cell-attached recordings the external solution served as pipette solution and agonists/UBP684 were include in this solution. In case of perforated whole-cell patch-clamp recordings the pipette solution was supplemented with 20 µg/ml of gramicidin. Whole-cell configuration after giga-ohm seal was reached typically within 10–15 minutes. Recordings were obtained using a Axopatch 200B amplifier (Axon Instruments/Molecular Devices) and digitized with pCLAMP 10 software (Axon Instruments/Molecular Devices). Whole-cell recordings were obtained at −70 mV, filtered at 2 kHz, and digitized at 5 kHz. For cell-attached recordings a potential (V_m_) of +70 mV was applied and data were filtered at 5 kHz (−3 dB, 8-pole Bessel) and digitized at 20 kHz. Single-channel amplitude was not corrected for junction potential.

The internal solution used for whole-cell recordings consisted of (in mM) 110 cesium gluconate, 30 CsCl_2_, 5 HEPES, 4 NaCl, 0.5 CaCl_2_, 2 MgCl_2_, 5 BAPTA, 2 Na_2_ATP, and 0.3 Na_2_GTP (pH 7.3). Rapid perfusion for whole-cell concentration jumps was achieved with a two-barreled theta glass pipette controlled by a piezoelectric translator (Burleigh). The solution exchange times for 10–90% solution were typically ~1–2 ms for open tip. Thus for the whole-cell the complete exchange of solution with take a few milliseconds. Thus the exchange time is not a limiting factor to determine the deactivation time constants. Cell was exposed to control solution with no drugs followed rapidly by a solution containing agonists ± UBP684. Drug application was typically for 2.5 s during each 15 s sweep.

### Data processing and kinetic modeling

Upward deflections were considered as open state during cell-attached recordings. Recordings containing a single active channel were idealized using QUB software (www.qub.buffalo.edu) as previously described^23, 35^. In addition to lack of visual observation of two simultaneous opening, to confirm the presence of only one active channel in our patches we utilized an approximation method^36^ that we have previously detailed using GluN1/GluN2C receptors which have a very lower open probability (0.011)^23^. Based on this approximation we are confident that the patches utilized in our studies contained only one active channel. The idealized data was used for maximum interval likelihood fitting (MIL; ref. 36). A 120 μs dead time was imposed using QUB. The C5O2 model consisting of 3 closed and 2 open states in linear configuration and two desensitized states emerging from C1 and C2 respectively provided the best fit to the single channel data based on the log likelihood values compared to other models including the C4O2 model with four instead of five closed states and a model where the receptor can transition to either a fast or slow gating step as described previously^35^. The mean open time, mean shut time and open probability was obtained from the idealized data using ChanneLab (www.synaptosoft.com), with an imposed dead time of 120 μs. Peak and steady state responses and deactivation, and desensitization time course for whole-cell recordings were analyzed using Clampfit (pClamp 10.2).

### Macroscopic response fitting

For macroscopic fitting, whole-cell responses to glutamate application on cells pre-equilibrated with glycine or glycine + UBP684 were obtained. The average control and UBP684 wave forms were normalized with the peak currents to be equal to the respective open probabilities (Fig. 3). Aggregated Markov models were fitted to individual wave forms using a nonlinear least sum of squares algorithm (ChanneLab). Glutamate binding and unbinding rates and desensitization rates were set as free parameters and the other gating steps were fixed to those obtained using MIL fits to single-channel data (Fig. 4).

### Statistical Analysis

All values are expressed as mean ± SEM. Data were compared using paired *t*-test for macroscopic current profiles and unpaired *t*-test and one-way ANOVA for the cell-attached patches. Values of P ≤ 0.05 were considered significantly different.

### Data availability

All relevant data are available from the authors.
